# The efficacy and safety of totally laparoscopic hepatectomy for non-cirrhotic hepatocellular carcinoma in the elderly

**DOI:** 10.1186/s12893-018-0444-x

**Published:** 2018-12-17

**Authors:** Xin Yu, Yan Chun Yan, Gang Chen, Hong Yu

**Affiliations:** 1 Department of Anaesthesiology, Sir Run Run Shaw Hospital, School of Medicine, Zhejiang University, Hangzhou, China; 2 Department of General Surgery, Sir Run Run Shaw Hospital, School of Medicine, Zhejiang University, Institute of Minimally Invasive Surgery of Zhejiang University, Key laboratory of laparoscopic technique of Zhejiang province, Qingchun Road 3, Hangzhou, 310016 China

**Keywords:** Laparoscopic hepatectomy, Elderly patients, Hepatocellular carcinoma

## Abstract

**Background:**

Laparoscopic hepatectomy (LH) has been identified to be effective and safe for elderly patients (≥70 years). This study aims to assess the short-and long-term outcome of totally laparoscopic liver resection for elderly patients with Hepatocellular carcinoma (HCC).

**Methods:**

We retrospectively reviewed 93 patients with HCC who underwent LH from August, 2003 to July, 2013 in a single center. Short-term operative and postoperative outcomes together with long-term outcomes, including disease free survival (DFS) and overall survival (OS) were analyzed.

**Results:**

A total of 81 patients was finally reviewed, of which 23 patients (28.40%) were grouped to elderly (≥70 years) and 58 patients (71.60%) were divided into younger group (< 70 years). The mean ages of patients in the elderly and younger cohorts were 74.9 ± 3.4 and 50.9 ± 12.7 years old, respectively. The median follow-up durations in elderly cohort and young cohort were 30 months and 24 months. The mean postoperative hospital stay was nearly 4 days longer in the elderly cohort than that in younger group (13.4 vs 9.5; *p* = 0.003). The elderly cohort has a higher rate of non-surgical complications than that in the younger cohort (*P* = 0.045), while the risks of surgical complications were comparable between the two groups. For the postoperative complications, elderly patients were more easily to develop grade III or more of Clavien-Dindo classification than that in the younger patients (*P* = 0.008). The median OS in the elderly group and younger group was 44.09 months and 42.49 months, respectively, with *p* = 0.089. The median DFS in the elderly group and the younger group was 39.87 months and 37.86 months, respectively, with *p* = 0.0616.

**Conclusions:**

Elderly patients could obtain comparable operative and survival benefits from LH for HCC as younger counterparts. Age may not be a contraindication to laparoscopic liver resection for elderly patients.

## Background

Hepatocellular carcinoma (HCC) is the most common malignant tumor of primary liver cancer and is the second leading cause of death from cancer worldwide [1]. Older age is widely considered to be a risk factor for HCC [2] and it has been reported an anticipated 67% increase in cancer incidence among patients older than 65 years from 2010 to 2030 compared with an 11% increase for youngers, and among those cancers, the incidence of hepatobiliary malignancies among patients more than 65 years old would be above 88% [3]. Due to the lifetime accumulation of different diseases, elderly patients always have been considered as clinically fragile, thus some effective therapies of HCC are only applied to the younger people. There is still lack of evidence to prove whether these useful treatments are also benefit to elderly patients. Therefore, it is becoming a regular clinical issue to manage the dramatically increasing elderly patients with HCC.

Surgical resection is considered a first-line curative option for HCC [4, 5]. However, many structural and functional changes can be induced in liver by aging, which could reduce the tolerability of hepatectomy, including the decreased mass of functional hepatocytes, decline in liver volume, as well as alterations in hepatic microcirculation. Thus, lots of elderly patients fear of not getting survival advantage and do not take the optimal therapy for HCC [6]. Now laparoscopic hepatectomy (LH) for liver cancers as a minimally invasive treatment has been showed to be effective and safe, with less blood loss, shorter hospital stay, fewer postoperative complications, and comparable survival [7, 8]. However, the majority of studies examined cohorts affected by a variety of benign and malignant pathologies, without specific focus on patients over 70 years for HCC. Whether the benefit of LH in elderly patient similar as in young patients still remains unclear. To solve this clinical problem, we designed this retrospective study to evaluate efficacy and safety of LH in elderly patients by investigating the short- and long-term surgical outcomes of LH in elderly HCC patients.

## Methods

This study had obtained the ethics committee from University Health Network and Sir Run Run Shaw Hospital. We retrospectively reviewed 93 patients who underwent LH for HCC in Sir Run Run Shaw Hospital of ZheJiang University from August,1998 to July, 2013. All surgery operations of these 93 patients were executed by a same team of experienced hepatobiliary surgeons (CaiXiuJun team, 3 Attending surgeons). Twelve patients were excluded as five patients lost following visits and seven patients converted to open procedures.

### Inclusion and exclusion criteria

LH was the initial therapy for all patients in this study for primary liver tumors. The indication of performing LH for liver malignancies included the tumor size smaller than 10 cm, without tumors invading major vessels, Child-Plug score no worse than B, absence of tumor thrombus in the main portal vein, as well as anatomically suitable and technically feasible. The diagnosis of HCC was confirmed by histologic examination after resection. Patients who were younger than 18 years old, ASA > IV or shift to open procedures were excluded.

### Short-term outcomes and complications

Operative factors including blood loss, blood transfusion, extent of liver resection and operative time and postoperative complications were recorded. As Brisbane 2000 terminology of liver anatomy and resections described [9], minor liver resection was defined as removal of one or two segments, while major liver resection referred to three or more segments removed. Postoperative complications consisting of surgery related complications and non-surgery related complication were also analyzed. Dindo-Clavien classification was used to classify postoperative complications [10]. Grade III or higher grade complications were considered as severe. If the patient occurred more than two complications, only the highest one would be considered in the data analysis. Indocyanine green clearance (ICG) with retention at 15 min of < 15% was considered adequate reserve and routinely assessed to represent liver function [11].

### Long-term outcomes

Length of postoperative hospital stay, overall survival (OS) rate, as well as disease free survival (DFS) rate were examined. Death within 90 days of LH was considered as perioperative mortality.

### Surgical technique

Laparoscopic liver resection was conducted with patients in the Lloyd-Davis position and with the surgeon standing between patients’ legs. If the mass located in Segments V and VI for a right hepatectomy, patients would be positioned in a wedge-shaped cushion with the table turned to its left side. Four trocars were inserted and actual placements of working ports depended on the location of the mass. A carbon dioxide pneumoperitoneum was established and maintained no more than 15 mmHg. Liver parenchymal transection was performed with LPMOD (Peng’s multifunction operative dissector, SY-IIIB, Hangzhou ShuYou Medical Equipment Co., Ltd., China) which was not only able to cut and coagulate the tissues, but also suction the blood and smoke to provide a clear view. Regional occlusion of liver left/right inflow and outflow instead of total hepatic vascular occlusion was applied to minimize ischemia reperfusion injury [12]. The resected specimens were put into a protective bag and pulled out by dilated incision (6 - 8 cm). The raw surface was carefully coagulated inch by inch to prevent active bleeding and bile leakage. A drainage tube was placed near the transection plane.

If there were uncontrolled bleeding, unclear tumor margin, severe adhesion, embolism or other complications occurred, laparoscopic procedure would be changed to open hepatectomy. The converted cases were excluded in the analysis.

After discharged from hospital, all patients had been followed up monthly within the first year. The examinations included physical examinations, computed tomographic scan or magnetic resonance imaging scan, and alpha-feto-protein (AFP). If no recurrence was detected, they would extend the follow up to quarterly. Recurrence was defined as new typical features of mass on imaging, or a rising AFP level. Biopsy was performed when necessary.

### Statistical analysis

Continuous data were expressed as median (range) or mean (standard deviation). The differences between elderly group and younger group were analyzed using Student’s *t* test or nonparametric Mann-Whitney test. Categorical variables were described as percentages. The Chi-square test was conducted to detect differences. Disease-free survival rate and overall survival rate were estimated using Kaplan–Meier analysis. A two-sided *P* < 0.05 was regarded as statistically significant. All statistical analyses were performed using the SPSS 13.0.

## Results

Ninety-three patients were retrospectively reviewed, five patients lost visiting and seven patients converted to open procedures. Among conversion patients, 2 patients (8.70%) were ≥ 70 and 5 patients (8.62%) were <  70 years old. Thus, a total of 81 patients were included in the analysis, 23 patients (28.4%) were ≥ 70 and 58 patients (71.6%) were <  70 years old. The basic demographics and tumor characteristics between elderly patients and younger patients are summarized in Table 1. The mean ages of patients in the elderly and younger cohorts were 74.9 ± 3.4 and 50.9 ± 12.7 years old, respectively. Other variables, including gender, body mass index, liver function, Child-Pugh score, tumor size, tumor number and the tumor stage, have no significant difference between the two groups. The elderly group had a greater prevalence of comorbidity and more frequently ASA score of 3.

**Table 1:** Demographics and Tumor characteristics of patients

Variable	Group A (≥ 70 y) (*N* = 23)	Group B (< 70 y) (*N* = 58)	*p*-value
Age (years) mean ± SD	74.9 ± 3.4	50.9 ± 12.7	0.003*
Gender (M/F)	21/2	46/12	0.329
BMI (kg/m^2^) mean ± SD	22.7 ± 3.1	22.3 ± 2.9	0.586
ASA score			
I/II	16	53	0.137
III	7 (30.4%)	5(9.4%)	0.047
ICGR 15(%) mean ± SD	11.8 ± 4.5	10.3 ± 3.9	0.129
Album g/dl	3.65 ± 0.5	3.84 ± 0.48	0.231
Total bilirubin mg/d	0.83 ± 0.5	0.87 ± 0.46	0.625
Comorbidity (present/absent)			
Cardiac diseases *n* (%)	4 (17.4)	1 (1.7)	0.041*
Diabetes *n* (%)	2 (8.7)	4 (6.9)	0.780
Hypertension *n* (%)	7 (30.4)	6 (10.3)	0.551
COPD *n* (%)	3 (13.0)	1 (1.7)	0.067
Cirrhosis *n* (%)	4 (17.4)	12 (20.6)	0.078
Child-Pugh classification A/B/C	91.3/ 8.7/0	82.8/ 17.2/0	0.493
HBV n (%)	12 (52.2)	37 (63.8)	0.450
Tumor size (cm)	4.7 ± 2.0	3.7 ± 2.1	0.403
Tumor size ≥5 cm *n* (%)	7 (30.4)	14 (24.1)	0.582
Tumor number 1/2/≥3 (%)	95.7/ 4.3/ 0	87.9/ 8.6/ 3.4	0.520
UICC7			0.882
Stage I	12 (52.17%)	37(63.79%)	
Stage II	5 (21.73%)	6(10.34%)	
Stage IIIA	5(21.73%)	12(20.69%)	
Stage IIIB	1(4.34%)	2(3.45%)	
Stage IIIC	0	1(1.72%)	
Previous abdominal operation *n* (%)	10 (43.5)	16 (27.6)	0.193
Extent of live resection			0.388
Wedge *n* (%)	4 (17.39)	18 (31.03)	
Segmentectomy *n* (%)	15(65.21)	28(50.90)	
Hemihepatectomy *n* (%)	4 (17.39)	12(20.68)	

*HCC* Hepatocellular carcinoma, *BMI* body mass index, *ASA* American Society of Anesthesiology

*: *P* value < 0.05

The median diameter of the largest tumor was 4.7 ± 2.0 cm and 3.7 ± 2.1 cm in elderly group and younger group, respectively. The median number of tumors was 1 (range, 1–3) and 1 (range, 1–5) in elderly group and younger group, respectively.

### Short-term outcomes and complications

Table 2 displays the perioperative and postoperative outcomes of the two groups. The median blood loss and the need of blood transfusion had no differences between the two groups (490 ml vs 401 ml, *P* = 0.434; 1.7% vs 17.2%, *P* = 0.752). Notably, the mean postoperative hospital stay was nearly 4 days longer in the elderly cohort when compared to that in younger patients group (13.4 vs 9.5; *p* = 0.003). The overall complication rate in elderly group was almost as similar as younger group. However, in detail, the elderly cohort has significantly higher rate of non-surgical complications than that in the young cohort (*P* = 0.045), while the risks of surgical complications were comparable between the two groups. In elderly cohort group, pneumonia was the most frequent non-surgical complications, and then followed by ascites, atrial fibrillation as well as liver failure. For the postoperative complications, elderly patients were more easily to develop grade III or more of Clavien-Dindo classification than that in the younger patients (*P* = 0.008).

**Table 2:** Perioperative and postoperative outcomes

Variable	Group A (≥ 70 y) (*N* = 23)	Group B (< 70 y) (*N* = 58)	*P*-value
Blood loss (ml)	490.0 ± 443.7	401.4 ± 462.2	0.434
Blood transfusion	5 (21.7)	10 (17.2)	0.752
Extent of liver resection (Major/ Minor)	4/19	12/46	0.737
Operative time (min)	135.4 ± 74.2	128.3 ± 50.3	0.696
Ovellrall	9(39.13)	13(22.42)	0.127
Surgical complication	4 (17.39)	9 (15.52)	0.836
Bile leak *n* (%)	1 (4.34)	3 (5.17)	
Intraabdominal sepsis *n* (%)	2 (8.70)	3 (5.17)	
Pleural effusion *n* (%)	0	2 (3.45)	
Surgical site infection *n* (%)	0	1 (1.72)	
Bleeding *n* (%)	1 (4.35)	0	
Non-surgical complication *n* (%)	5 (21.74)	4 (6.90)	0.045
Pneumonia *n* (%)	2 (8.70)	0	
Ascites n (%)	1 (4.35)	3 (5.17)	
Atrial fibrillation *n* (%)	1 (4.35)	1 (1.72)	
Liver failure *n* (%)	1 (4.35)	1 (1.72)	
90-days mobidity	0	0	
Clavien classfication			
Grade I-II	6	10	0.367
Grade III-V	4	1	0.008*
Recurrence time months	32.9 ± 23.7	34.3 ± 33.6	0.721
TACE *n* (%)	13 (56.5)	28 (48.3)	0.624
Postoperative complications *n* (%)	8 (34.8)	11 (19.0)	0.152
Postoperative hospital stay (d)	13.4 ± 7.5	9.5 ± 4.0	0.003*

*TACE* transarterial chemoembolization (TACE)

### Long-term outcome

**Fig. 1:**
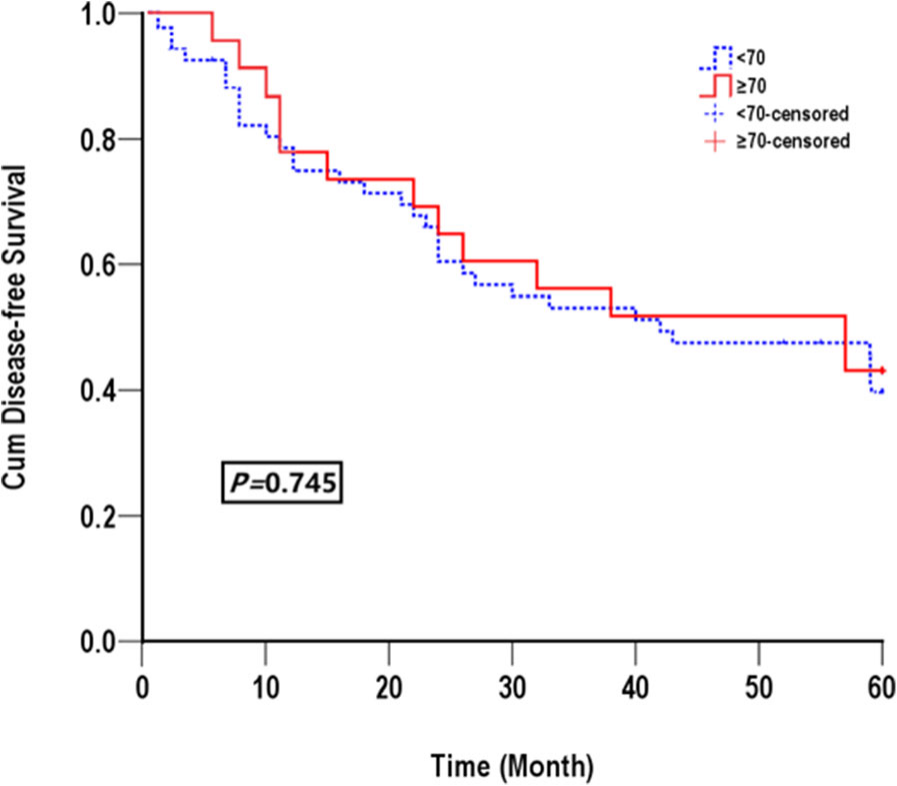
Disease-free survival for all patients in elderly group (solid line) and in younger group (dashed line)

**Fig. 2:**
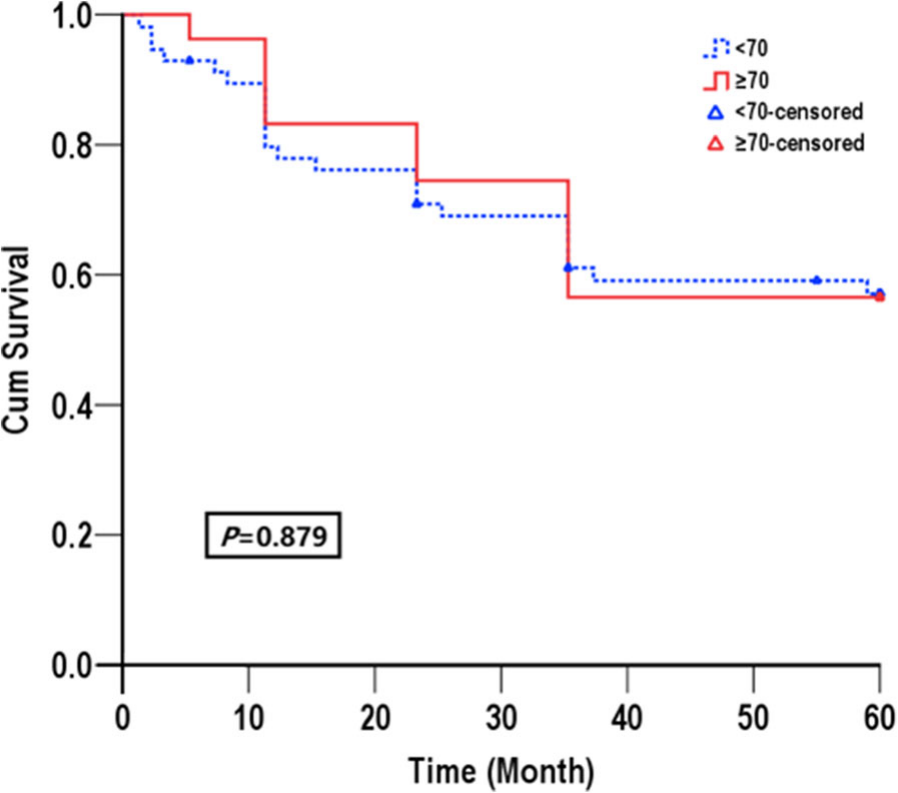
Overall survival for all patients in elderly group (solid line) and in younger group (dashed line)

The median follow-up durations in elderly cohort and young cohort were 30 months and 24 months, respectively. The DFS and OS of elderly and younger group are showed in Table 3. The median DFS rate in the elderly group and the younger group was 39.87 (95%CI: 31.75–43.98) months and 37.86 (95%CI: 33.49–45.69) months, respectively. The cumulative rate of 1, 3, and 5 year DFS were 78.26, 52.17 and 43.48% in the elderly group, which were as similar as in the younger group (75.28,51.58 and 39.91%, respectively). There was no significant difference of DFS between the two groups (*p* = 0.745) (Fig. 1). Likewise, the median OS was 44.09 (95%CI: 36.05–52.13) months in the elderly group and 42.49 (95%CI: 36.33–46.34) months in the younger group, and no significant difference was found between the two groups (*P* = 0.879). The cumulative rate of 1, 3, and 5-year OS were 82.61, 73.91, and 56.52% in the elderly group, compared to 79.11, 61.07, and 57.06% in the younger group (Fig. 2).

**Table 3:** Overall and disease free survival in the two groups

	Medium months	95%CI	1Y(%)	3Y(%)	5Y(%)	*p*-value
Disease free survival						*P* = 0.745
≥ 70 year old	39.87	31.75~ 43.98	78.26	52.17	43.48	
< 70 years	37.86	33.49~ 45.69	75.28	51.58	39.91	
Overall survival						*P* = 0.879
≥ 70 year old	44.09	36.05~ 52.13	82.61	73.91	56.52	
< 70 years	42.49	36.63~ 48.34	79.11	61.07	57.06

## Discussion

To our knowledge, the safety and survival study of laparoscopic liver resection on elderly is relative rare. So far, this study has been the largest study which assessed both short-term and long-term outcomes after LH for HCC.

In our study, we found that big difference in some features existed between elderly and younger patients. Firstly, the mean age in elderly group was almost 20 years older than younger group. Elderly patients were predominated by man. The proportion of elderly in this study was consistence with previous unselected study [13]. Secondly, the two groups had different distribution in concomitant diseases and ASA III class. As shown in Table 1, elderly had higher rate of cardiac diseases (17.4%) and respiratory disorders (13%). On the other hand, there were no statistical differences between the two groups in terms of the conversion rate, tumor stage, tumor number and size, intraoperative blood loss, as well as blood transfusion. Two patients in the elderly group died of heart disease which may due to their preoperative cardiovascular diseases.

In this study, we found that postoperative overall complications and surgical complications rates had no statistical difference between the elderly cohort and younger cohort (39.13% vs 22.42, 17.39% vs 15.52%). The comparable surgical complications could be contributed to the lack of abdominal surgery and its corresponding consequences. However, the non-surgical complications like pneumonia, atrial vibration, and liver failure were more frequent in elderly patients comparing with that in younger group (21.74% vs 6.9% *P* = 0.045), which may result from the expected significant difference in ASA class in the elderly cohort who had decreased physical organ functioning and physical activity. Thus, when performing LH in elderly, more attention should be paid on physical status, comorbidities, and organ functionalities rather than age.

Previous studies reported that liver resection via the open approach in the elderly (65 to 70 years) was very high mortality range from 3.5 to 5.6% [14, 15]. However, our results showed zero perioperative mortality rate of LH in elderly, which may associate with decreased blood loss and decreased surgical wall trauma with laparoscopic approach. In our study, 17.39% of the elderly patients underwent hemihepatectomy and none of them experienced mortality, which may also lead to the zero-mortality rate. Because major hepatectomy increases the perioperation mortality [16, 17]. Furtherly, in our population, the rate of blood loss and blood transfusion was low. The result was consistent with published studies, providing more evidence to prove the efficiency of LH [18].

We also found that the elderly patients had a longer postoperative hospital stay than that of the younger patients (13.4 ± 7.5 vs 9.5 ± 4.0, *P* = 0.003). Limited functional reserve and lower recovery capacity in the elderly patients may delay the hospital leave. Recently, advances in postoperative pain administration and early postoperative rehabilitation in our center have improved the cardiopulmonary function recovery and reduced the hospital stay, which bring promising perspective for HCC patients, especially for the elderly. However, this topic is still controversial as those advanced techniques were found to be age-independent [19].

Whether the outcome of HCC treated by LH is affected by age is the key issue that this study needs to address, hence the disease-free survival and overall survival were also analyzed. We found that elderly patients and younger patients had a similar medium survival time (44.09 M vs 42.49 M). Meanwhile, the overall survival in 1-year, 3-year and 5-year had no big difference in both group (*p* = 0.879). The reason may be due to the low survival rate of both group (< 60% at 5 years). Despite a higher frequency, ASA III score and 10 years older in elderly group, the life expectancy of elderly patients was not influenced. Previous study by Fong and colleagues showed that the worse basic disease conditions and ASA grade were risky predictors of poor clinical outcomes, however these factors only affect cardiopulmonary complications and have little effect on cancer occurrence and overall survival [20]. There was no significant difference between the two groups regarding to medium disease free survival time DFS (39.87 M vs 37.86 M). Interestingly, a trend toward better DFS in elderly group was observed (43.48% vs 39.91%, *p* = 0.745), which may due to that tumors in elderly patients were less aggressive than those in younger patients.

## Conclusion

LH takes advantages than open procedure not only in the rapid recovery but also in the long-term prognosis. In our study, the 5-year OS and DFS with LH for HCC was higher than those in open procedures [21]. The better results in the LH group can be explained by 2 main reasons. First is less blood loss in LH, which is a risk factor for HCC recurrence [22]. Secondly, “no-touch” technique in LH is also associated with better oncological outcomes [23]. In HCC patients, venous permeation and vascular invasion are responsible for preoperative hematogenous spread of tumor cells. Open hepatectomy may compress tumor in the condition of mobilization and then enhance tumor cells to spread into intrahepatic portal venous system or even circulation.

Several limitations of this study must be considered, such as the retrospective analysis and limited size. The elderly operated group might suffer from selection bias for non-operated of minimal invasively treatment population was missing. Furthermore, the results of this study only came from a single specialized center, so the results may not generalize to the whole population.

We found that there were no big differences in blood loss, postoperative complication, disease-free survival and overall survival for LH between the elderly patients and younger patients. So, appropriate selected elderly patients can obtain comparable operative and survival benefits from LH for HCC as younger counterparts. Performing surgical procedures in elderly patients, more emphasis should be paid on physical status, organ function and comorbidities rather than his/her age.

## Abbreviations

AFP: Alpha-feto-protein
DFS: Disease free survival
HCC: Hepatocellular carcinoma
ICG: Indocyanine green clearance
LH: Laparoscopic hepatectomy
LPMOD: Peng’s multifunction operative dissector
OS: Overall survival
